# Towards the second stage—*Journal of Intensive Care*

**DOI:** 10.1186/s40560-018-0282-z

**Published:** 2018-03-06

**Authors:** Hiroshi Morisaki

**Affiliations:** Department of Anesthesiology, Keio University School of Medicine, 35 Shinanomachi, Shinjuku-ku, Tokyo, 160-8582 Japan

**Fig. 1:**
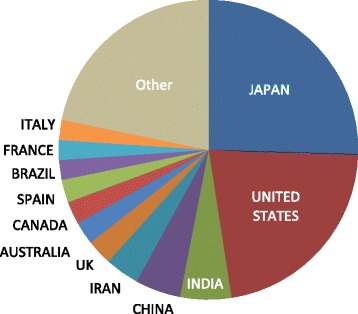
2017 Submissions by country

It is my great honor and concurrently challenging task to make this statement as the new *Editor-in-chief* of the *Journal of Intensive Care*. To date, this *Journal*, which was launched in October 2013, as the official journal of the *Japanese Society of Intensive Care Medicine* (JSICM), has been outstandingly led by Professor Satoshi Gando, the founding Editor-in-chief. Being an international journal, the members of Advisory and Editorial Board embrace many worldwide leaders in the intensive care field. Based on their continuous and persevering efforts, in addition to the enthusiastic contribution by all authors and/or researchers, we are proud of a number of remarkable and significant articles published in the first stage of this newborn journal. In the first 2 years after its launch, more than 55% of the submissions came from Japanese researchers and/or intensivists; now, almost 75% of the submissions come from researchers in other areas of the world, including North America and Europe, as well as Asia, Middle East, and Oceania (Fig. 1).

**Fig. 2:**
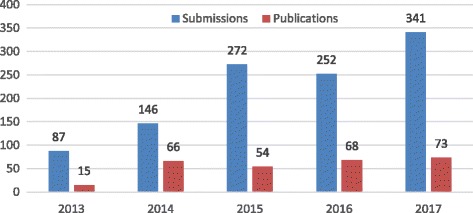
Submissions and publications

Recently, there has been a notable increase in the number of submissions. Last year, the *Journal of Intensive Care* received 341 submissions, which amounts to an approximately 35% increase in comparison to the previous year (Fig. 2). Predictably, the overall acceptance rate is decreased to 21.3% last year, while 19.7% of research articles submitted were finally accepted for publication. Furthermore, I dare to declare that we have continued to accept the submission of case reports on cases showing an extraordinary clinical course in which the discussion is informative to the readers; however, the acceptance rate of *case reports* is currently 7.3%. It should also be noted that the average intervals from the formal receipt of a manuscript to the dates of the first and final decisions were 16.7 and 33.4 days, respectively. Although nobody can conclude whether the current editorial and reviewing processes are short enough to satisfy the authors/researchers, I believe that both high-quality reviewing and prompt publication are indispensable for a medical journal. On behalf of the *Editorial Board Members*, I would like to express my sincere appreciation to all peer reviewers for their persistent, patient, and noble contribution to this Journal.

Owing to the countless journals that have been launched in the last decade, international journals must now compete with each other within the same field of medicine. By the end of 2017, there were 57 and 137 journals including the term, intensive care and critical care, respectively. To prevail and thereby step forward in these types of struggles between the journals, we must continue to work hard on improving both the academic and clinical quality of this journal by publishing high-quality articles and simultaneously providing more constructive peer-review processes. To date, all articles published in the *Journal of Intensive Care* are indexed in worldwide services such as DOAJ, PubMed, PubMed Central, and Scopus. Now, based on continuous and overall assessment, our journal has been uploaded on the Emerging Sources Citation Index (ESCI), implying that the journal is close to obtaining Social Sciences Citations Index value (i.e., impact factor).

*Towards the Second Stage* as an international journal, the *Journal of Intensive Care* shall work hard to contribute to the development of state-of-the-art intensive care and to thereby amend both the short- and long-term outcomes of critically ill patients. Our Editorial Board members earnestly look forward to a number of submissions from all over the world that will further improve the state of intensive care better than today.

